# Supplementary material for: A Perspective on the CD47-SIRPA Axis in High-Risk Neuroblastoma
Source: Curr Oncol. 2024 Jun 1;31(6):3212–26. doi: 10.3390/curroncol31060243 (PMC11202629; doi:10.3390/curroncol31060243)
Supplement: Supplementary file 1 [file curroncol-31-00243-s001.zip › curroncol-3019171-supplementary.pdf]

Supplemental materials

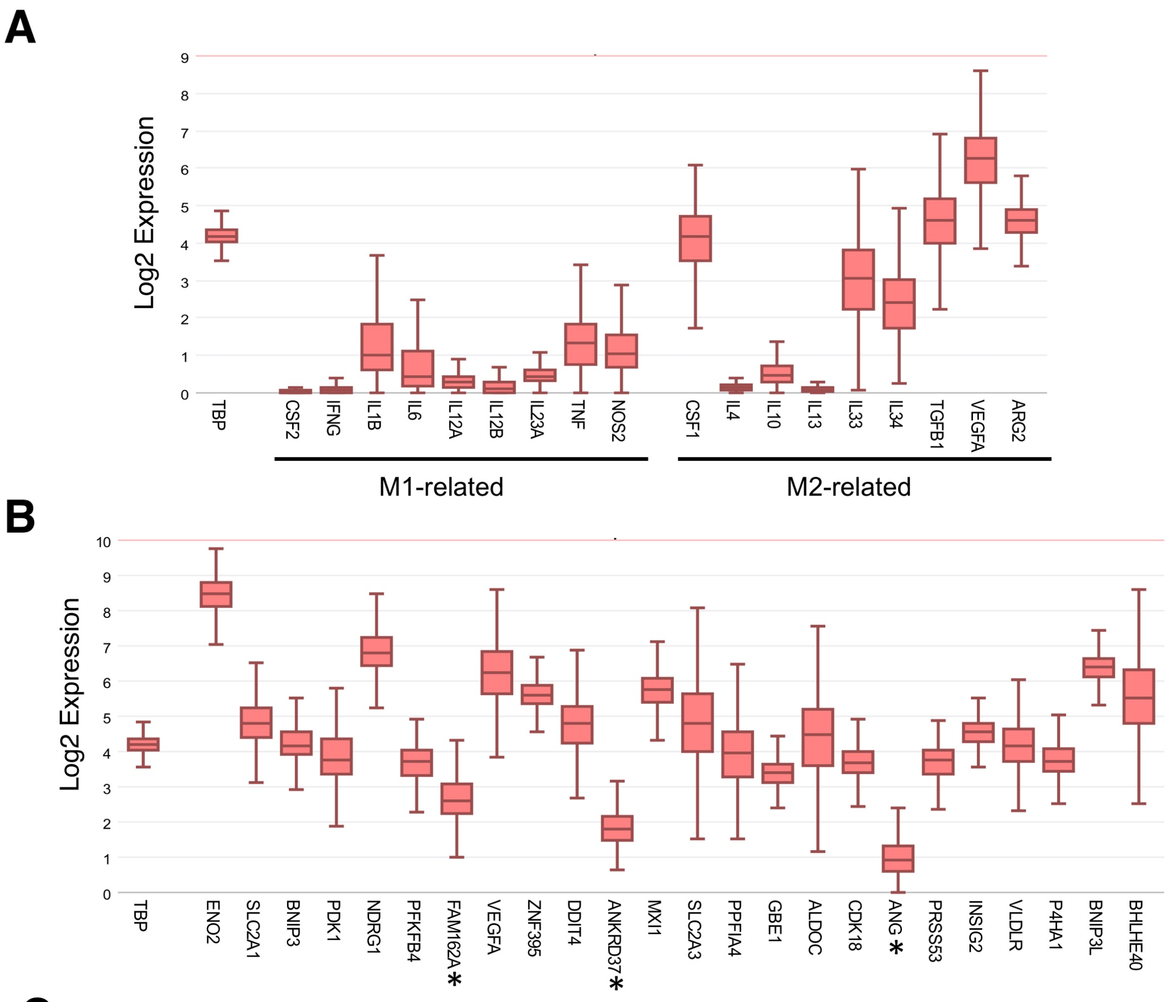

C

Correlation of *CD68* and *CD163* gene expressions with phagocytosis and immune cell activation marker gene expressions in high-risk neuroblastomas*		
	CD68	***CD163***
**Efferocytosis**		
CD300LB	r= 0.693, p=1.75e-26	r= 0.593, p=4.54e-18
MERTK	r= 0.775, p=1.46e-36	r= 0.824, p=8.72e-45
STAB1	r= 0.720, p=1.94e-29	r= 0.760, p=1.98e-34
**Phagocytosis**		
CD11B	r= 0.884, p=1.90e-59	r= 0.819, p=8.17e-44
CD18	r= 0.932, p=1.83e-78	r= 0.830, p=6.55e-46
SLAMF7	r= 0.808, p=7.97e-42	r= 0.642, p=7.19e-22
LRP1	r= 0.543, p=6.57e-15	r= 0.531, p=3.49e-14
FCGR2A	r= 0.840, p=5.06e-48	r= 0.873, p=3.38e-56
FCGR3A	r= 0.713, p=1.20e-28	r= 0.813, p=1.11e-42
**Phagosome formation**		
RAC2	r= 0.685, p=1.02e-25	r= 0.558, p=9.10e-16
RHOG	r= 0.685, p=1.02e-25	r= 0.558, p=9.10e-16
VAV1	r= 0.833, p=1.55e-46	r= 0.726, p=4.11e-30
**Macrophage activation**		
CD38	r= 0.767, p=2.26e-35	r= 0.656, p=4.83e-23
CXCL10	r= 0.681, p=2.59e-25	r=0.590, p=7.16e-18
*The high-risk subset of GSE62564 dataset (*n*=176)		

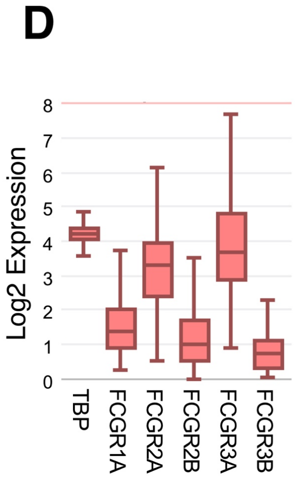

Figure S1. Macrophage polarization and phagocytic capacity in high-risk neuroblastoma tissues. (A) The expression of genes encoding cytokines, lymphokines and enzymes for bioresponse modification that can influence the polarization of TAMs in high-risk neuroblastoma tissues. Expression levels of M2-related genes (*CSF1*, *IL33*, *IL34*, *TGFB1*, *VEGFA, ARG2*) were higher than those of M1-related genes (*IL1B*, *IL6*, *TNF, NOS2*). (B) The expression of hypoxia signature genes (19) in high-risk neuroblastoma tissues. Among the twenty-four hypoxia signature genes examined, there were twenty-one genes that were expressed at elevated levels in high-risk neuroblastoma. These include *ENO2*, *SLC2A1*, *BNIP3*, *PDK1*, *NDRG1*, *PFKFB4*, *VEGFA*, *ZNF395*, *DDIT4*, *MXI1*, *SLC2A3*, *PPFIA4*, *GBE1*, *ALDOC*, *CDK18*, *PRSS53*, *INSIG2*, *VLDLR*, *P4HA1*, *BNIP3L*, and *BHLHE40*. The three genes that were expressed at low levels were *FAM162A*, *ANKRD37*, and *ANG* (indicated by asterisks). (C) Correlation of *CD68* and *CD163* gene expressions with phagocytosis related and macrophage-activation marker genes. Efferocytosis is a distinctive form of phagocytosis for the removal of apoptotic cells, mediated by phosphatidylserine receptors, such as *CD300LB* (*CD300B*), *MERTK*, and *STAB1*, which are highly expressed on macrophages (Human Protein Atlas) (62). Efferocytosis of apoptotic cells leads to M2 polarization of macrophages (83, 84). Other phagocytosis related genes include *CD11B/CD18*, *SLAMF7* and *LRP1*. SLAMF7 synergizes with Mac-1 (CD11B/CD18) to recognize ligands on target cells (85). *RAC2, RHOH* and *VAV1* are preferentially expressed in macrophages (Human Protein Atlas)) (62), and their products are involved in phagosome formation. The expression of *CD38* and *CXCL10* genes signifies the activation status of macrophages in tumor tissues (22, 23). (D) *FCGR* expressions in high-risk neuroblastoma tissues. In the context of antibody-based immunotherapy targeting macrophages, Fcγ receptors play the crucial role in ADCP. Among genes encoding activating Fcγ receptors (FCGR1A, FCGR2A and FCGR3A) and inhibitory receptors (FCGR2B and FCGR3B), *FCGR2A* and *FCGR3A* are dominant *FCGR* genes expressed in high-risk neuroblastoma tissues and their expression was significantly corelated with *CD68* and *CD163* (shown in **C**). The R2: Genomics Analysis and Visualization Platform (http://r2.amc.nl) (43) was used for gene expression profiling analyses on the high-risk neuroblastoma cohort (*n*=176) of the SEQC dataset (NCBI GEO, GSE62564) collected at diagnosis (17, 18). The housekeeping gene *TBP,* encoding TATA box binding protein, was used as the reference gene.

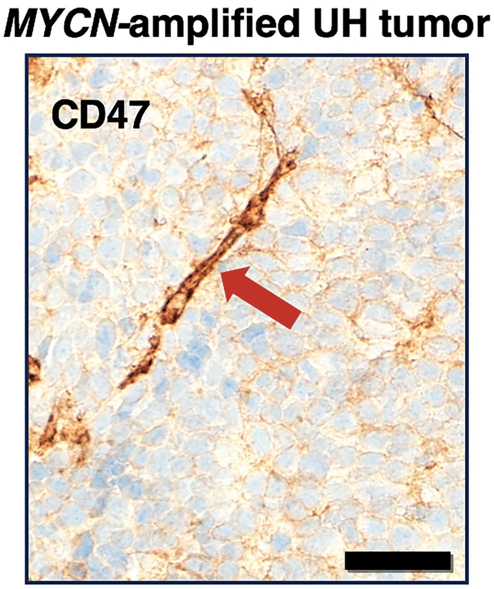

Figure S2. CD47 expression on vascular cells of high-risk neuroblastoma tissues. Immunohistochemical analysis reveals CD47 staining on the vasculature (indicated by the red arrow) of a high-risk neuroblastoma with *MYCN* amplification. Scale bar: 20μm.

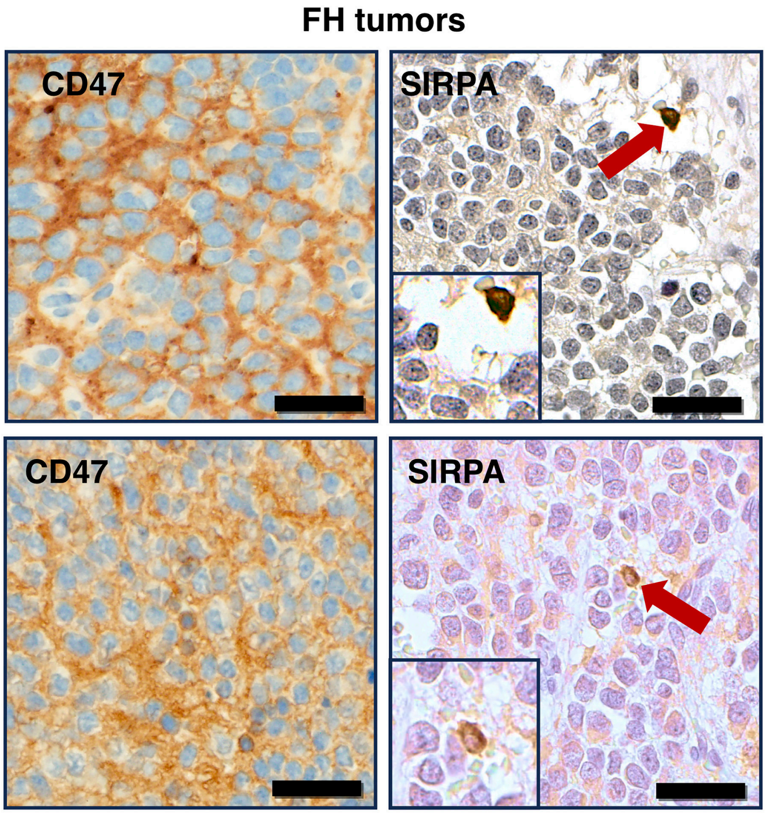

Figure S3. CD47 and SIRPA expression in favorable neuroblastoma. Immunohistochemistry was performed on three favorable neuroblastoma specimens using anti-CD47 and anti-SIRPA antibodies indicated in Figure 1. Representative images are presented. Weak cell membrane staining of CD47 and strong CD47 staining on neurites of the favorable tumor cells were observed. Myeloid cells with SIRPA expression (red arrows) were sparsely present in tumor tissues. Based on the cell size, they are likely early/differentiating macrophages. Insets show images of macrophages with 2-fold enlargement of the original. FH: Favorable Histology. Scale bars: 20μm.

Table S1. Anti-SIRPα antibodies tested in human clinical trials for malignant diseases

**Name**<br>**Synonyms**	**Ref**	**IgG type***<br>**Fc type****	**Target**	**Trial Phase†**	**Clinical Trial ID**
ADU-1805	(70)	IgG2	Solid Tumor, Adult<br>Metastatic Solid Tumor<br>Refractory Cancer	Phase 1<br>Recruiting	NCT05856981
BI-770371	(71)	IgG1	Solid Tumors	Phase 1<br>Recruiting	NCT05327946
			Carcinoma, Squamous Cell of Head and Neck<br>Melanoma<br>Non-small Cell Lung Cancer	Phase 1<br>Recruiting	NCT05068102
BI-765063<br>OSE-172	(86)	IgG4<br>S229P-L445P	Solid Tumor, Adult	Phase 1<br>Active, not recruiting	NCT03990233
			Head and Neck Squamous Cell Carcinoma	Phase 1<br>Active, not recruiting	NCT05249426
BR-105	(68)	IgG1<br>N297A	Neoplasms	Phase 1<br>Not yet recruiting	NCT05351697
BYON-4228	(87)	IgG1<br>L234A/L235A	Lymphoma	Phase 1<br>Recruiting	NCT05737628
CC-95251<br>BMS-986351<br>Anzurstobart	(69)	IgG1<br>K322A	Neoplasms	Phase 1<br>Active, not recruiting	NCT03783403
			Leukemia, Myeloid, Acute<br>Myelodysplastic Syndrome	Phase 1<br>Active, not recruiting	NCT05168202
DS-1103a	(88)	IgG4	Advanced Solid Tumor<br>Breast Cancer	Phase 1<br>Recruiting	NCT05765851

*IgG1 has high affinity for FcγRs; IgG2 and IgG4 have low affinity for FcγRs (75).
** IgG4, S229P-L445P: increasing stability (89, 90); IgG1, N297A: reducing FcγR binding (72); IgG1, L234A/L235A: abolishing FcγR and C1q binding; IgG1, K322A: reducing FcγR binding and abolishing C1q binding (91).
†As of April 11, 2024.

References

See the main text.
